# Artificial Induction of Associative Olfactory Memory by Optogenetic and Thermogenetic Activation of Olfactory Sensory Neurons and Octopaminergic Neurons in *Drosophila* Larvae

**DOI:** 10.3389/fnbeh.2016.00137

**Published:** 2016-06-28

**Authors:** Takato Honda, Chi-Yu Lee, Ken Honjo, Katsuo Furukubo-Tokunaga

**Affiliations:** ^1^Institute of Biological Sciences, University of TsukubaTsukuba, Japan; ^2^Ph.D. Program in Human Biology, School of Integrative and Global Majors, University of TsukubaTsukuba, Japan; ^3^International Institute for Integrative Sleep Medicine (WPI-IIIS), University of TsukubaTsukuba, Japan

**Keywords:** *Drosophila*, learning and memory, behavior assay, optogenetics, thermogenetics

## Abstract

The larval brain of *Drosophila melanogaster* provides an excellent system for the study of the neurocircuitry mechanism of memory. Recent development of neurogenetic techniques in fruit flies enables manipulations of neuronal activities in freely behaving animals. This protocol describes detailed steps for artificial induction of olfactory associative memory in *Drosophila* larvae. In this protocol, the natural reward signal is substituted by thermogenetic activation of octopaminergic neurons in the brain. In parallel, the odor signal is substituted by optogenetic activation of a specific class of olfactory receptor neurons. Association of reward and odor stimuli is achieved with the concomitant application of blue light and heat that leads to activation of both sets of neurons in living transgenic larvae. Given its operational simplicity and robustness, this method could be utilized to further our knowledge on the neurocircuitry mechanism of memory in the fly brain.

## Introduction

In the past decades, the development of powerful neurogenetic techniques in the fruit fly *Drosophila melanogaster* has contributed to further studies on the molecular and cellular mechanisms of learning and memory (Heisenberg, 2003; Davis, 2011; Guven-Ozkan and Davis, 2014). In particular, with simple and identifiable neural networks, *Drosophila* larvae provide an ideal system for the elucidation of the underlying neurocircuitry mechanism (Aceves-Piña and Quinn, 1979; Heisenberg et al., 1985; Tully et al., 1994; Vosshall and Stocker, 2007; Furukubo-Tokunaga and Hirth, 2012; Diegelmann et al., 2013). Previous works showed that associative olfactory memory could be induced in larvae either by the two-odor reciprocal protocol (Scherer et al., 2003; Hendel et al., 2005; Michels et al., 2005; Selcho et al., 2009, 2014; Gerber et al., 2010; Diegelmann et al., 2013) or by the single-odor nonreciprocal protocol (Honjo and Furukubo-Tokunaga, 2005, 2009; Khurana et al., 2009; Kleber et al., 2016). While many studies have utilized the two-odor reciprocal protocol, it involves several intricate handling steps (Hendel et al., 2005; Michels et al., 2005; Selcho et al., 2009, 2014; Pauls et al., 2010; Diegelmann et al., 2013). On the other hand, although care need to be taken for choosing controls, the single-odor protocol consists of fewer training cycles (Honjo and Furukubo-Tokunaga, 2005, 2009; Khurana et al., 2009; Pauls et al., 2010).

In this protocol, based on the singe-odor paradigm (Honjo and Furukubo-Tokunaga, 2005, 2009), we applied the optogenetic and thermogenetic techniques to induce associative olfactory memory in larvae without behavioral conditioning. Previous works showed that synaptic output from octopaminergic (OA) neurons is necessary and sufficient for appetitive memory formation in larvae (Schroll et al., 2006; Honjo and Furukubo-Tokunaga, 2009; Selcho et al., 2014). Signals from OA neurons are integrated with the olfactory information pathway at two prominent brain structures, the antennal lobes and mushroom bodies, to establish appetitive associative memory (Heisenberg, 2003; Davis, 2011; Furukubo-Tokunaga and Hirth, 2012; Guven-Ozkan and Davis, 2014). It was also shown that light-induced stimulation of olfactory receptor neurons (ORNs) induces olfactory response behaviors in living larvae (Bellmann et al., 2010; Störtkuhl and Fiala, 2011).

**Figure 1:**
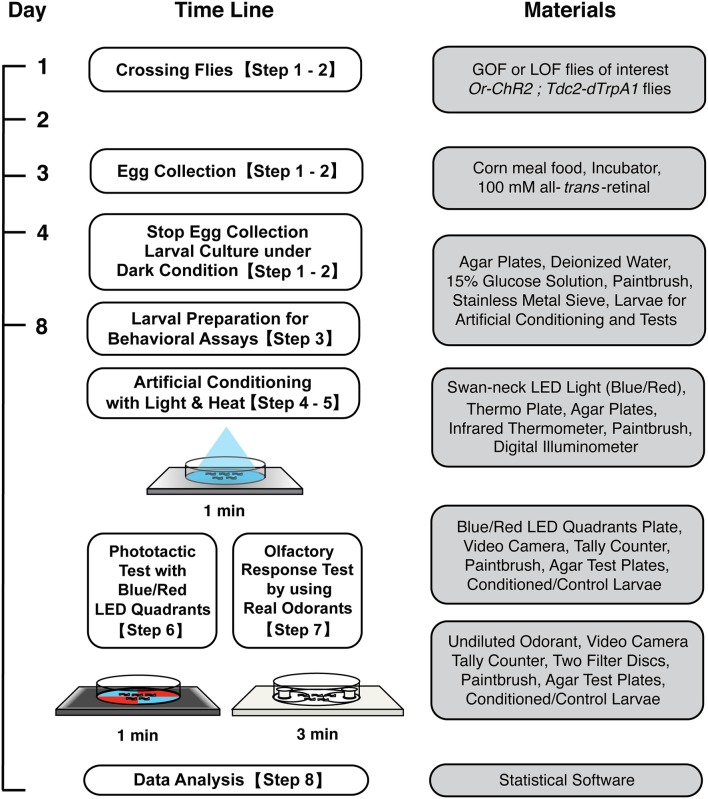
**Workflow for artificial induction of associative olfactory memory in ***Drosophila*** larvae**. After conditioning with blue light and heat, memory can be tested either on the blue/red LED quadrants plate or on an olfactory response test plate. See the text for detailed procedures.

**Figure 2:**
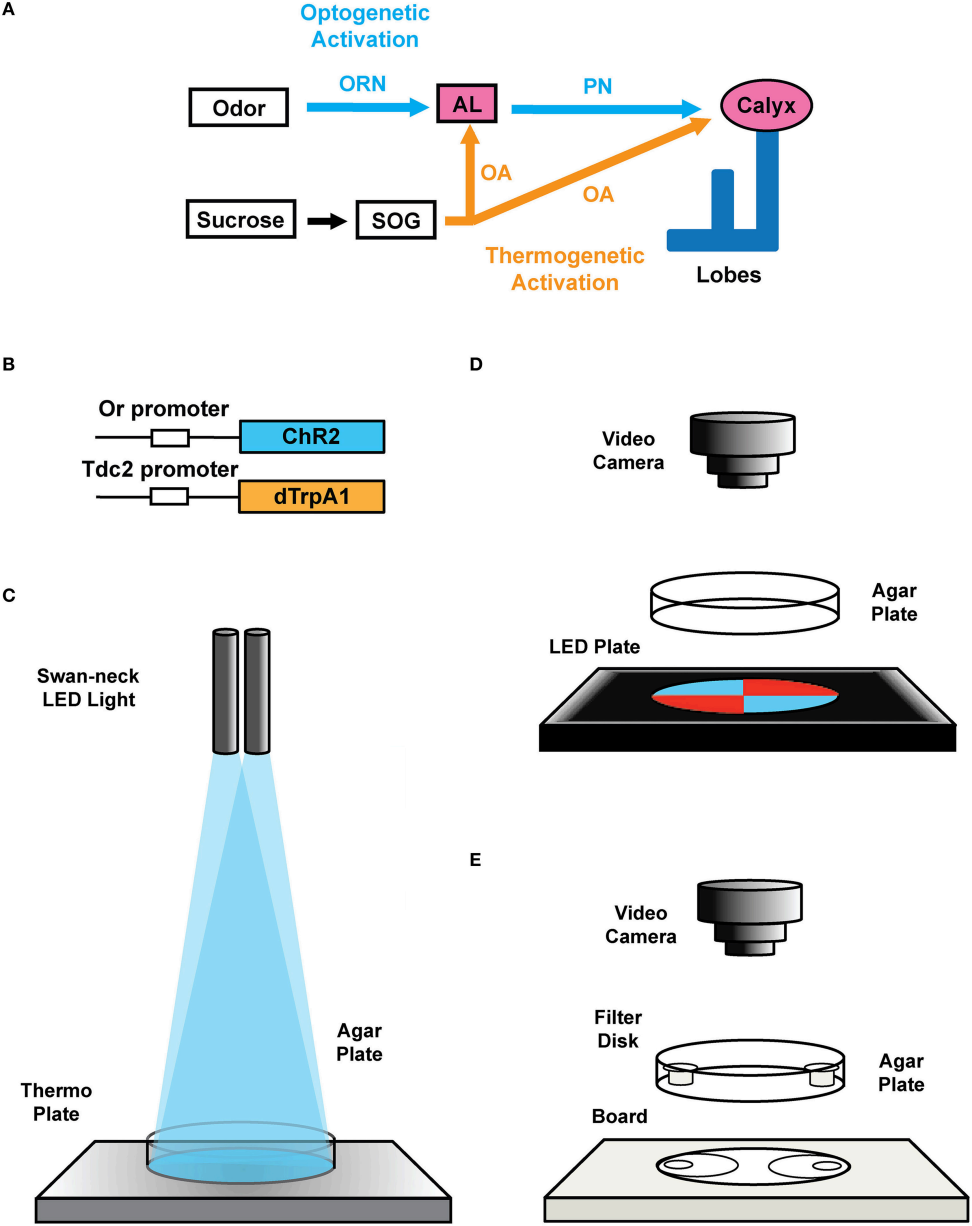
**Transgenic constructs and LED apparatus. (A)** Schematic diagram for artificial induction of associative olfactory memory in the fruit fly larvae. This method consists of (1) substitution of reward signals with dTrpA1-mediated thermogenetic activation of OA neurons and (2) substitution of the odor signals with ChR2-mediated optical activation of a specific class of ORNs. **(B)** Structures of the *Or-ChR2* and *Tdc2-dTrpA1* constructs. *Or-ChR2* construct; the coding sequence of ChR2 is directly placed under the transcriptional promoter sequence of an Or gene, which is expressed in a specific set of the larval ORNs. *Tdc2-dTrpA1* construct; the coding sequence of *dTrpA1* is fused with the transcriptional promoter sequence of the *Tdc2* gene, which is expressed in the larval OA neurons. **(C)** Setup for artificial conditioning with blue light and heat. Swan-neck LED light and thermo plate. **(D)** blue/red LED quadrants plate. **(E)** Olfactory response test plate.

Here, we describe step-by-step procedures of our protocol, which consists of (1) substitution of gustatory reward by thermogenetic activation of OA neurons with the *Drosophila* Transient receptor potential A1 (dTrpA1) channel (Hamada et al., 2008) under the control of the *tyrosine decarboxylase 2* (*Tdc2*) promoter, and (2) substitution of odor stimuli by optical activation of a specific class ORNs with Channelrhodopsin 2 (ChR2; Nagel et al., 2005), which is expressed under the control of a specific olfactory receptor (Or) promoter (Honda et al., 2014; Figures 1, 2A). After a brief paired conditioning with blue light and warm temperature, dual transgenic larvae (*Or-ChR2; Tdc2-dTrpA1*) exhibit significant suppression of negative phototaxis toward blue light. The unpaired presentation of blue light and heat causes no alteration in phototaxis behaviors, suggesting that associative memory is induced only by a concomitant activation of the converging set of the endogenous neurons involved in memory formation.

While conventional behavioral protocols involve multiple steps of larval preparation and conditioning by hands, this artificial activation protocol consists of minimum operational steps requiring only 60 s for memory induction. Despite this very short conditioning, olfactory memory produced by this protocol is robust and as stable as the memory produced with the combination of a natural odorant and reward. We also describe a light-based memory assay using a blue/red LED plate, which provides us with a sensitive alternative to the conventional olfactory test on an agar plate.

## Materials and equipment

All the materials and equipment used in this protocol are listed in Table 1.

**Table 1:** **Materials and equipments**.

**Name of material/equipment**	**Company**	**Catalog/model number**
All-trans-retinal	Sigma-Aldrich, MO, USA	R2500
Agar powder	Nacalai tesque, Tokyo, Japan	01028-85
Glucose	Sigma-Aldrich, MO, USA	G7021
100% Ethanol	Nacalai tesque, Tokyo, Japan	14713-95
Acetophenone	Nacalai tesque, Tokyo, Japan	00412-52
Ethyl acetate	Nacalai tesque, Tokyo, Japan	14747-65
Digital video camera	SONY, Tokyo, Japan	HDR-CX520
Digital illuminometer	AS ONE, Tokyo, Japan	AR-813A
Filter paper, Ø 70 mm (Cut and use in Ø 10 mm)	ADVANTEC, Tokyo, Japan	00021070
Petri dish, 90 × 15 mm	IWAKI, Tokyo, Japan	36-3412
Thermo Plate	TOKAI HIT, Tokyo, Japan	MATS-SPE
Twin LED light (Blue/Red)	RelyOn, Tokyo, Japan	Order-made
Infrared radiometer	Testo AG, Baden-Württemberg, Germany	830-T1
468 nm Blue LEDs (2600 mcd) with 490 nm short-path filter of 50% transmission	RelyOn, Tokyo, Japan	Order-made
625 nm Red LEDs (2600 mcd) with 580 nm long- path filter of 50% transmission	RelyOn, Tokyo, Japan	Order-made
GraphPad prism 6	GraphPad Software, CA, USA	MDF-GP6ECO

### Stepwise procedures

The workflow of this protocol is shown in Figure 1.

**1. Transgenic flies and lighting apparatus**

**Figure 3:**
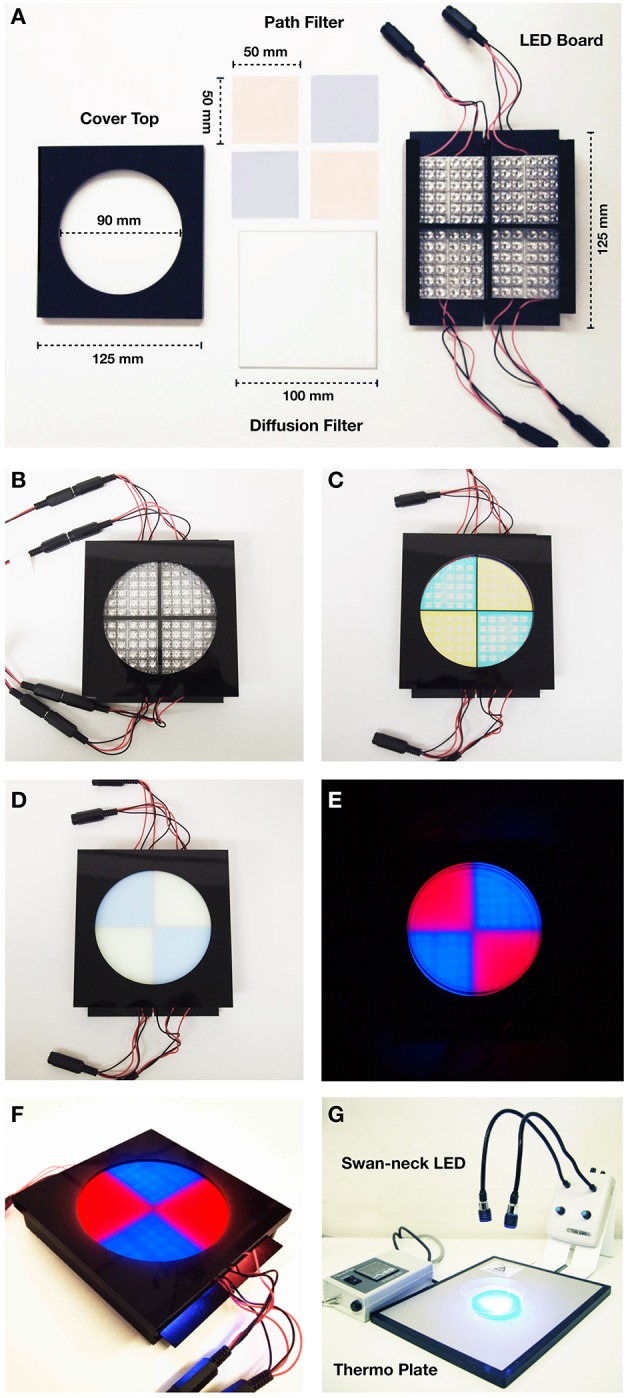
**Specification of Blue/Red LED device. (A)** Parts of the blue/red LED quadrants plate. (Left) cover top with a ∅ 90 mm hole; (upper middle) 490 nm short-path filters for 468 nm blue light LEDs and 580 nm long-path filters of for 625 nm red light LEDs; (lower middle) 100 mm × 100 mm diffusion filter of 50% transmission; (right) LED housing board. Each quadrant contains 36 LED units of 468 nm (2600 mcd) for blue light, and 625 nm (2600 mcd) for red light. **(B)** Set up with the cover exposing 22 LED units for each quadrant (14 units are covered). **(C)** Set up with the cover and the band-path filters. **(D)** Set up with the cover, the band-path filters, and the diffusion filter. **(E)** Photographic image of the blue/red LED test plate with agar plate on it. **(F)** Overview image of the blue/red LED test plate **(G)** Set up for conditioning: swan-neck LED light (468 nm blue light/635 nm red light) and thermo plate.

1.1. Description of the transgenic flies used in this protocol: In order to express ChR2 in a specific set of ORNs, we cloned the coding region of ChR2 under the specific promoter sequence of specific Or genes (Wang et al., 2003; Larsson et al., 2004; Fishilevich et al., 2005; Kreher et al., 2005, 2008; Or-ChR2 in Figure 2B). In order to express dTrpA1 in reward neurons (Hamada et al., 2008), we cloned the dTrpA1 coding sequence under the promoter of the *Tdc2* gene (Cole et al., 2005; *Tdc2-dTrpA1* in Figure 2B). See the construct design described in the Supplemental Figure 1.
  Note: The Tdc2 promoter is known to drive specific expression in OA neurons in the larval brain (Honjo and Furukubo-Tokunaga, 2009; Selcho et al., 2012, 2014; Zhang et al., 2013). We have deposited these stocks at the Bloomington Stock Center (Indiana, USA). The standard stock used in this protocol is w (CS10), which was outcrossed with Canton S. All stocks were outcrossed to w (CS10) at least five times before experiments.
  Optional: Setting up genetic crossing with mutant flies. The transgenic larvae are homozygous for both *Or-ChR2* (2nd chromosome) and *Tdc2-dTrpA1* (third chromosome), which can be combined with either gain-of-function or loss-of function background by standard genetic crossing. Binary expression systems such as *GAL4-UAS* can be used to drive an overexpression or RNAi constructs in conjunction with this protocol (see Discussion).
1.2. Fly stock maintenance: Raise the flies with a standard corn meal food (5.5 g/L agar, 40 g/L yeast extract, 90 g/L cornmeal, 100 g/L glucose, and 0.7 g/L n-butyl-p-hydroxybenzoate). Maintain fly stocks in an incubator set to 25°C, 75% relative humidity and under dark or red dim light.
1.3. Lighting equipment: Set up a swan-neck lighting apparatus with light-emitting diodes (LEDs) (3 Watt each) on dual flexible arms (Figures 2C, 3G).
1.4. Blue/red LED quadrants plate: Prepare diametrically opposed quadrants, each illuminated with LEDs of 468 nm LED (2600 mcd) in the blue quadrants covered by a 490 nm short-path filter, and with 625 nm LED (2600 mcd) in the red quadrants covered with 580 nm long-path filter (Figures 2, 3). To ensure homogeneous illumination, diffusion filter of 50% transmission is placed on the LED units (Figure 3). The light intensity on the surface of the agar plate is approximately 2900 lux as measured with a digital illuminometer.
1.5. Agar plates: Make agar plates by dissolving 7.5 g agar powder in 300 mL deionized water (2.5% agar), heat up with a microwave and then pour 20 mL to each 90 mm petri dish. The agar plates can be kept at 4°C till the next day.

**2. Preparation of early third instar larvae**

2.1. Prepare 100 mM all-trans-retinal stock. Dissolve all-trans-retinal (100 mg) in 100% ethanol (3.52 mL). Aliquot the stock solution at 1 mL in 1.5 mL test tubes wrapped with aluminum foil, and store in a lightproof box at −20°C.
2.2. Make all-trans-retinal food. Melt corn meal food (10 mL corn meal food per vial) in a microwave oven, and add 100 μL all-trans-retinal (100 mM) per vial (final 1 mM). Mix well to ensure homogeneity.
2.3. Collect eggs for 4 h at 25°C under dark from adult flies of 3–10 days old. Do not include more than 50 flies in the vial to avoid overcrowding of emerging larvae.
2.4. Raise larvae in all-trans-retinal containing food at 25°C under dark for 72 h after the end of the egg collection (early third instar larvae).
  Note: We suggest using early third instar larvae (72–76 h after egg laying) because of easier handling compared to the earlier stages. Late third instar larvae could be used but care should be taken for staging to avoid the ecdysone effect.

**3. Harvesting larvae for conditioning**

3.1. All experiments on step 3–7 should be conducted at 25°C room temperature.
3.2. Preset the thermo plate at 36°C. Put an agar test plate on the thermo plate and check the surface temperature of agar to be 28°C with an infrared thermometer. Adjust the hot plate setting as required. Exact setting of the thermo plate may depend on the heating apparatus. Note also that it may take several minutes to have the equilibrium of the agar surface temperature.
3.3. Harvest early third instar larvae by pouring 15% glucose solution into vials, and gently disturb the food surface with a spatula to release larvae into the solution.
3.4. Collect the larvae on a stainless metal sieve (diameter 10 cm, aperture, 500 μm) by decantation. Remove food debris with forceps.
3.5. Transfer the larvae to 100 mL beaker by running distilled water from a washing bottle and using a paintbrush. Rinse the larvae three times gently with distilled water to remove food debris and glucose.
3.6. Transfer the larvae on a fine metal mesh (10 × 10 cm, aperture ~100 μm) with a paintbrush. Briefly touch paper towel on the other side of the mesh to remove the excess fluid.
3.7. Transfer the larvae to an agar plate (21°C) with a paintbrush. The prepared larvae can be kept on the agar up to 10 min before conditioning.

**4. Conditioning with blue light and heat**

4.1. Place a fresh agar plate on the thermo plate pre-adjusted in step 3.2. Ensure the surface temperature of agar is 28°C with an infrared thermometer.
4.2. Adjust the illumination of the swan-neck LED light (blue) to cover the entire surface of the agar plate (Figure 3G).
  Note: Set the light intensity on the agar surface at 8000 lux as measured with a digital illuminometer. Subsequent steps (4.3–6.2) should be conducted in the dark with a safe red light (~1 lux) for photographic work.
4.3. Gently place ~50 larvae on the pre-warmed agar plate with a paintbrush (Figure 2C).
4.4. Illuminate the animals for 60 s (continuous) with the swan-neck LED light (blue; Figures 2C, 3G).
  Note: Either longer (≥ 90 s) or shorter (≤ 45 s) illumination results in poor conditioning (Honda et al., 2014).

**5. Dissociation and light/heat control tests**

**Figure 4:**
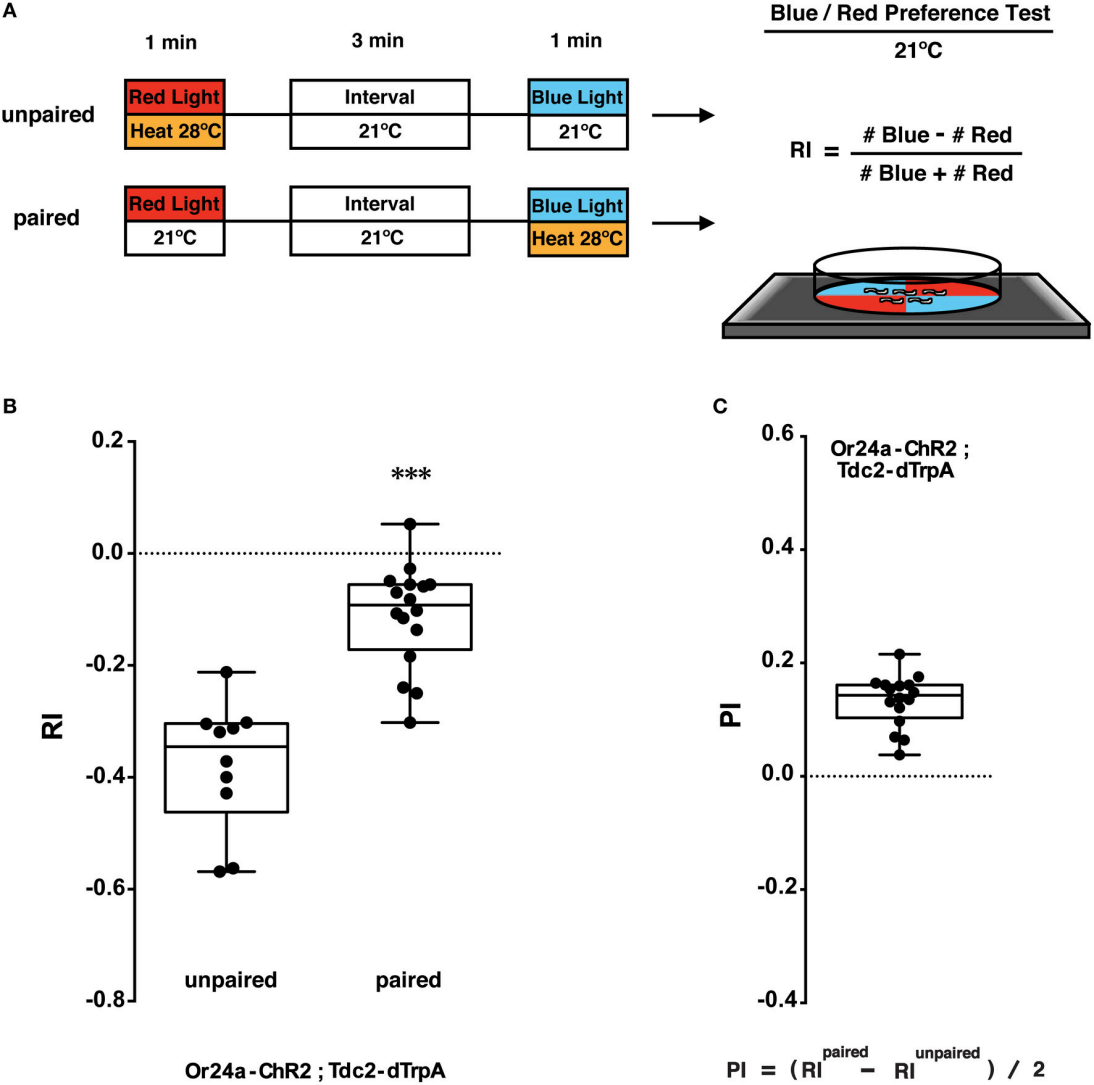
**Phototactic tests of larval memory formed with paired presentation of blue light and heat. (A)** Larval conditionings and phototaxis test. Transgenic larvae carrying both *Or-ChR2* and *Tdc2-dTrpA1* were conditioned with heat (28°C) and blue light in either unpaired or paired way. After the first conditioning with red light (60 s), larvae are transferred to an interval plate (21°C), and then transferred to another plate to be illuminated with blue light for 60 s. Heat stimulation was applied during either the first conditioning (unpaired) or the second conditioning (paired with blue light). Larval phototactic behavior was then tested at 21°C on the blue/red quadrants plate. **(B)** Phototactic responses of transgenic larvae (*Or24a-ChR2; Tdc2-dTrpA1*) with unpaired and paired conditioning. The paired but not the unpaired conditioning with blue light and heat (28°C) caused significant suppression of negative phototaxis toward blue light. ^***^p < 0.001 by Mann-Whitney *U*-test between unpaired and paired group. *n* = 10–16 trials. **(C)** Memory performance scores (PI) of the paired conditioning group against the unpaired control. Box plots represent the median as the middle line, 25th and 75th percentile as box boundaries, as well as the minimum and the maximum as whiskers, respectively.

**Figure 5:**
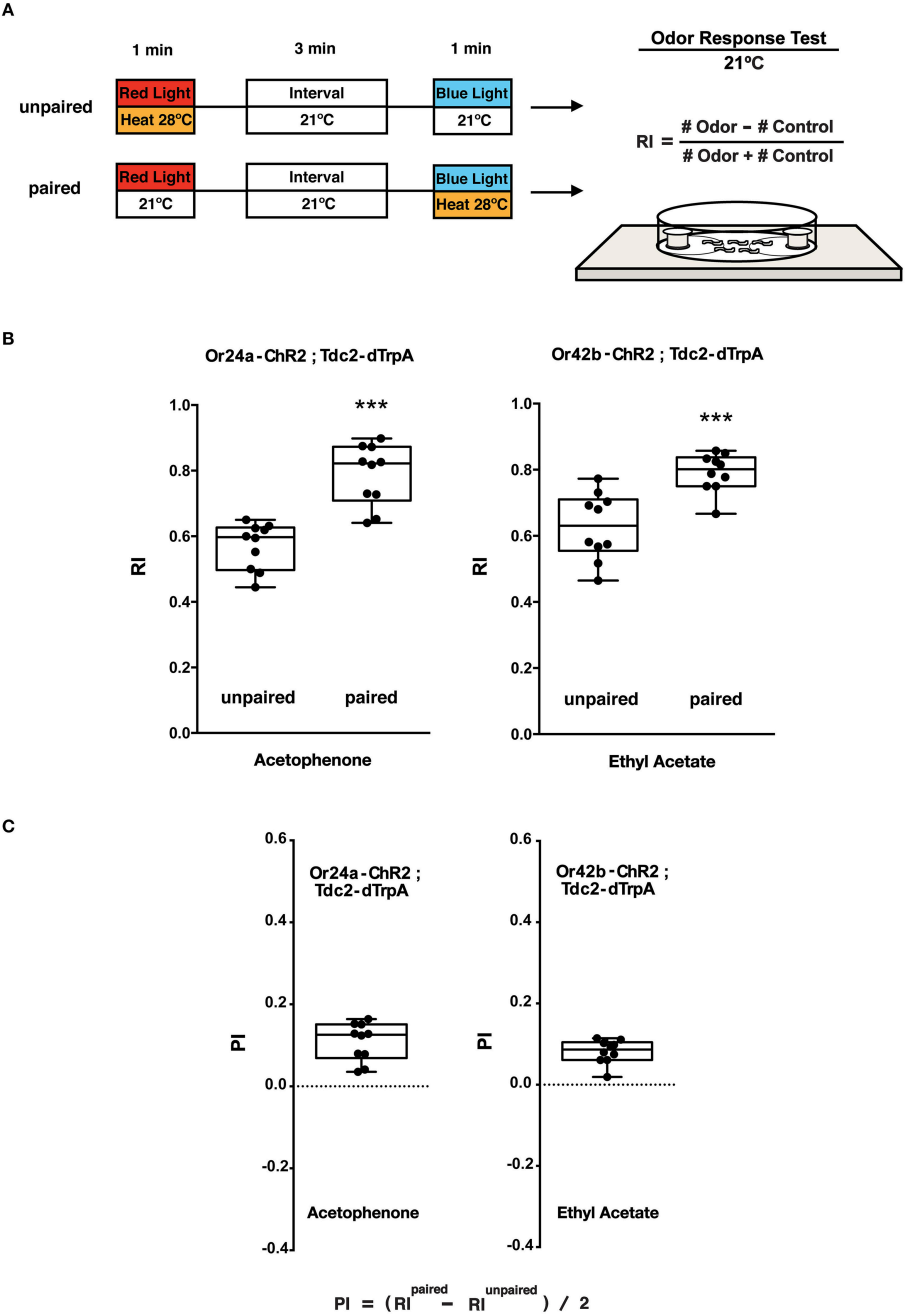
**Olfactory response tests of larval memory formed with paired presentation of blue light and heat. (A)** Larval conditionings and olfactory test. Transgenic larvae carrying both *Or-ChR2* and *Tdc2-dTrpA1* were conditioned with heat (28°C) and blue light in either unpaired or paired way as described in Figure 4A. Larval olfactory response was then tested at 21°C on the olfactory test plate. **(B)** Olfactory responses of transgenic larvae (*Or-ChR2; Tdc2-dTrpA1*) with unpaired and paired conditioning. The paired but not the unpaired conditioning with blue light and heat (28°C) caused significant increase in the olfactory response to the specific odorant determined by the *Or-ChR2* type. ^***^p < 0.001 by Mann-Whitney *U*-test between unpaired and paired group. *n* = 10 trials. **(C)** Memory performance scores (PI) of the paired conditioning group against the unpaired control. Box plots represent the median as the middle line, 25th and 75th percentile as box boundaries, as well as the minimum and the maximum as whiskers, respectively.

5.1. Dissociation tests: In order to verify associative conditioning, perform the following set of experiments, in which animals are exposed to both heat (28°C) and blue light but either unpaired or paired way (See the schematic diagrams in Figures 4A, 5A).
  (1) Place an agar plate on the thermo-plate and adjust the temperature of the agar surface either at 28°C (for unpaired conditioning) or at 21°C (for paired conditioning). Gently place ~50 larvae on the agar plate with a paintbrush. Illuminate the animals for 60 s with red light.
  (2) After the first conditioning, transfer the larvae to an agar plate pre-adjusted at 21°C. Keep the larvae for 3 min without light simulation.
  (3) After the interval, transfer the larvae to another agar plate pre-adjusted either at 21°C (for unpaired conditioning) or at 28°C (for paired conditioning), and illuminate them for 60 s with blue light.
  (4) Proceed to Step 6 or 7 for memory test at 21°C.
5.2. Light/heat control tests:
  To separately examine the effects of light and heat exposures, perform the following sets of conditioning (Supplemental Figure 2A) based on the procedures described in Step 4 but with different light/heat parameters. These experiments involve controls of basic larval handlings, which would help troubleshooting when the dissociation tests fail to work.
  $$\begin{array}{l} \text{Blue light at }21{}^{\circ}\text{C}\\ \text{Red light at }28{}^{\circ}\text{C}\\ \text{Red light at }21{}^{\circ}\text{C} \end{array}$$

**6. Larval phototaxis test with blue/red LED quadrants**

6.1. After conditioning, transfer the larvae with a paintbrush to the center of a fresh agar plate on the blue/red LED quadrants apparatus (Phototaxis test plate; Figures 2D, 3E), and leave the animals to choose the blue/red quadrants.
6.2. After 1 min, count the number of larvae in the blue and red quadrants. Exclude the larvae on the border of blue and red quadrants and the larvae crawling up the peripheral wall of the plastic plate from counting as they may be out of proper lighting.
6.3. Calculate the Response Index (RI) based on the following equation.
  $$\begin{array}{l} \text{RI}=\left({\text{N}}^{\text{Blue}}-{\text{N}}^{\text{Red}}\right)∕\left({\text{N}}^{\text{Blue}}+{\text{N}}^{\text{Red}}\right)\\ {\text{N}}^{\text{Blue}}:\text{The number of larvae in the blue quadrants}\\ {\text{N}}^{\text{Red}}:\text{The number of larvae in the red quadrants} \end{array}$$

**7. Olfactory Preference test**

7.1. Perform olfactory tests to verify odor-specificity and to exclude the possibility of general desensitization toward blue light (Figure 5A and Supplemental Figure 3A). The olfactory response test described here is based on the agar plate assay described previously (Aceves-Piña and Quinn, 1979; Rodrigues, 1980; Monte et al., 1989), and can be conducted under normal light condition (~400 lux).
  Note: Some of the odorants are potentially harmful and flammable. They should be dispensed to a small quantity (10 μL) in the fume hood before experiments.
7.2. Prepare a fresh agar plate (2.5%, 90 mm). Place a lid of a 1.5 mL microcentrifuge tube on both sides, 5 mm away from the outer edge (Figure 2E and Supplemental Figure 3A), and put paper filter discs (10 mm diameter) on them. Avoid touching the lid and filter discs with bare hands.
7.3. After conditioning with blue light and heat (step 4), transfer ~50 animals to the center of the test plate with a paintbrush (Figure 5A and Supplemental Figure 3A). Spread tangled animals gently with a paintbrush. Spot 2.5 μL undiluted odorant on the test disc and none on the control disc. Close the lid immediately.
7.4. After 3 min, count the number of larvae inside of the semicircular areas (3 cm in radius from each filter disc).
7.5. Calculate the RI based on the following equation.
  $$\begin{array}{l} \text{RI}=\left({\text{N}}^{\text{Odor}}-{\text{N}}^{\text{Control}}\right)∕\left({\text{N}}^{\text{Odor}}+{\text{N}}^{\text{Control}}\right)\\ {\text{N}}^{\text{Odor}}:\text{The number of larvae in the odor area}\\ {\text{N}}^{\text{Control}}:\text{The number of larvae in the control area} \end{array}$$

**8. Data analysis**

8.1. Calculation of memory performance scores: To analyze the extent of behavioral changes, calculate the memory performance score based on the following equation (Kleber et al., 2016; Figures 4C, 5C).
  $$\begin{array}{l} \text{Performance Index}=\left({\text{RI}}^{\text{Paired}}-{\text{RI}}^{\text{Unpaired}}\right)∕2 \end{array}$$
8.2. Statistics: Perform power calculation to determine the exact sample size (number of trials) necessary for the sufficient effect size and statistical power. This would require performing larval memory tests at least 10 times.
8.3. Examination of data distribution: Since the obtained Response Index (RI) is not always normally distributed, normality tests such as the D'Agostino-Pearson (Omnibus K2) test should be performed against the obtained data.
  If the data set shows a Gaussian distribution (*P* ≥ 0.05 in normality tests), perform parametric test such as Student's *t*-test (for pair-wise comparisons) and ANOVA followed by Tukey's *post-hoc* test (for multiple comparisons). If the data set fails to show a Gaussian distribution, perform non-parametric tests such as Mann-Whitney U-test (for pair-wise comparisons) and Kruskal-Wallis test followed by Dunn's *post-hoc* test (for multiple comparisons).

## Anticipated results

### Memory test with blue/red LED quadrants

The larvae were stimulated with blue light and heat (28°C) in unpaired and paired ways (Figure 4A). Concomitant stimulation with blue light and heat caused significant suppression of negative phototaxis toward blue light as compared to unpaired exposure to both stimuli (*p* < 0.001, Figure 4B). A comparison between paired- and unpaired-trained groups revealed significant associative memory with the positive PI value (Figure 4C). Associative memory performance was assessed using the equation (RI^Paired^ − RI^Unpaired^)/2 (Kleber et al., 2016). These results showed that associative memory was induced only by a concomitant activation of the converging set of the endogenous neurons involved in memory formation.

As light/heat controls, animals were stimulated for 60 s with either blue or red light in combination with 21 or 28°C (light/heat controls; Supplemental Figure 2A). The phototactic behavior of the dual transgenic larvae (*Or-ChR2; Tdc2-dTrpA1*) was not altered by red light at either temperature (Supplemental Figure 2B). By contrast, blue light stimulation at 28°C but not at 21°C caused significant suppression of negative phototaxis toward blue light (*p* < 0.001, Supplemental Figure 3B). No difference on RI was observed for the *w (CS10)* control larvae by either conditioning (Supplemental Figure 2B).

### Memory test with specific odorants

The animals were stimulated with blue light and heat (28°C) in unpaired and paired ways (Figure 5A). Only the paired stimulation with blue light and heat transgenic larvae (*Or24a-ChR2; Tdc2-dTrpA1* and *Or42b-ChR2; Tdc2-dTrpA1*) resulted in significant increases in the olfactory response toward the specific odorant, acetophenone or ethyl acetate, respectively (*p* < 0.001, Figures 5B,C). These results demonstrated that the artificial memory was specific to the odorant determined by the type of the ORNs stimulated with blue light.

Compared to the control (60 s blue light at 21°C), associative conditioning (60 s blue light at 28°C) of transgenic larvae resulted in significant increases in the olfactory response toward the odorant predicted by the *Or-ChR2* type (*p* < 0.01, Supplemental Figure 3B). Transgenic larvae carrying *Or24a-ChR2* showed increased response toward acetophenone while transgenic larvae carrying *Or42b-ChR2* showed increased response toward ethyl acetate (Supplemental Figure 3B).

Statistical calculations were performed using Prism 6 (GraphPad, San Diego, CA, USA). Further examples of experimental results can be found in Honda et al. (2014).

## Discussion

Various techniques have been published for olfactory associative learning in *Drosophila* larvae (Aceves-Piña and Quinn, 1979; Heisenberg et al., 1985; Tully et al., 1994; Scherer et al., 2003; Schwaerzel et al., 2003; Hendel et al., 2005; Honjo and Furukubo-Tokunaga, 2005; Michels et al., 2005; Vosshall and Stocker, 2007; Honjo and Furukubo-Tokunaga, 2009; Khurana et al., 2009; Selcho et al., 2009; Gerber et al., 2010; Furukubo-Tokunaga and Hirth, 2012; Diegelmann et al., 2013; Selcho et al., 2014; Kleber et al., 2016). It has been shown that *TßH* mutant larvae exhibit abnormal locomotor behaviors (Saraswati et al., 2004; Selcho et al., 2012), and that ablation of the *Tdc2* neurons in the ventral nervous system impairs larval locomotion (Selcho et al., 2012). However, in contrast to the locomotor deficits caused by chronic inactivation, acute activation of the *Tdc2* neurons (60 s) during conditioning did not alter the larval phototactic and olfactory responses in our protocol unless it was paired with the activation of the Or neurons (Figures 4, 5). These results were also confirmed by the light/heat control data (Supplemental Figure 2B), which showed that 60 s heat stimulation under red light did not alter larval response, and consistent with the results by Schroll et al., which show that temporal light activation of the OA/tyramine neurons did not impair odor perception and locomotor activity (Schroll et al., 2006).

### Significance with respect to existing methods

Unlike the conventional behavioral protocols, our protocol selectively stimulates the target neurons for memory induction, and thus significantly reduces the background neuronal activities. It is also noteworthy that conventional animal training with natural odorants leads to activation of multiple ORN classes due to the redundancy in Or affinities to diverse odors (Kreher et al., 2005, 2008; Hallem et al., 2006), which generates complex olfactory codes in the memory centers. By contrast, stimulating only a single type ORNs, the protocol described in this work provides us with a highly simplified setup for the olfactory code, which would be of help for high-resolution analyses of neural circuits involved in memory dynamics in the fly brain.

In addition, since animals are conditioned remotely, this technique can be adapted for memory experiments under the microscope using dissected brains. In such cases, formation of associative memory could be identified with optical activity reporters, such as GCaMP (Broussard et al., 2014) or CaMPARI (Fosque et al., 2015), expressed in the memory centers in the brain. Furthermore, because the transgenic larvae used in this technique do not involve the *GAL4-UAS* system, it allows complementary usage of various binary expression systems including not only the *GAL4-UAS* but also other expression systems such as *LexA-LexAop* or *QF-QUAS* (del Valle Rodríguez et al., 2012) either to monitor the activities of other neurons or to manipulate additional sets of neurons.

Lilly and Carlson (1990) showed that *Drosophila* larvae are negatively phototactic and partition onto the dark quadrants on the phototactic assay plate. We found similar negative phototaxis on the blue-red quadrants, where naïve larvae avoid blue light. Despite this innate behavior, we found that the phototaxis assay on the blue/red LED quadrants is highly sensitive to detect the behavioral alterations after the artificial conditioning by this protocol.

While we have utilized the *Tdc2* promoter to drive dTrpA1 in OA/tyramine neurons, recent studies have shown that different sets of dopaminergic neurons mediate positive and negative values in the adult flies (Aso et al., 2010, 2012; Burke et al., 2012; Liu et al., 2012). It has been shown that the D1-DA receptor dDA1 is required for both aversive and appetitive learning in larvae (Kim et al., 2007; Selcho et al., 2009). Whereas the *TH-GAL4* driver, which includes the PPL1 but not the PAM cluster neurons, fails to mediates reward leaning in larvae (Honjo and Furukubo-Tokunaga, 2009), a recent work (Rohwedder et al., 2016) has shown that reward value is mediated by PAM-like dopamine neurons (pPAM) in the larval brain. Direct activation of larval PAM neurons would be an attractive choice in future experiments.

### Critical steps and troubleshooting

One of the most critical steps is the preparation of larvae in good conditions. Overcrowded cultures should not be used since they are likely to produce poorly behaving animals (Step 2.3). For efficient ChR2 stimulation, all-*trans*-retinal (final 1 mM) needs to be supplemented in the larval culture food (Step 2.2) (see also Honjo et al. (2012). Details of larval handling are also described in Monte et al. (1989).

Heating at high temperature (>30°C) is noxious for larvae (Tracey et al., 2003). Precise measurement of the plate temperature using an infrared thermometer is recommended (Step 3.2 and 4.1). On the other hand, negative control experiments for dTrpA1 stimulation should be performed at 21°C, which is low enough to silence the channel. Associative conditioning with blue light is best achieved with ~1 min illumination (Step 4.4). Longer illumination may lead to poor memory scores (Honda et al., 2014).

For both conditioning and response tests (Step 4–7), it is important not to apply more than 50 larvae on the plate to avoid excess animal tangling, which alters subsequent larval behavioral responses. Gentle spreading of tangled larvae is of help.

To ensure the combined effect of light and heat, we suggest performing the dissociation test (Step 5.1, Figures 4, 5; Honjo and Furukubo-Tokunaga, 2005; Kleber et al., 2016). These experiments involve stimulation of the transgenic larvae with both heat and blue light but in unpaired and paired ways, and thus essential to verify the associative effect of the unconditioned and conditioned stimuli.

For technical controls and troubleshooting, we suggest performing the light/heat controls (Step 5.2, Supplemental Figure 2), which examine the individual effect of heat and blue light. It also provides a control of basic larval handling without heat and ChR2 stimulation (red light at 21°C).

### Limitations of the protocol

This protocol is designed for *Drosophila* larvae carrying both the *Or-ChR2* and *Tdc2-dTrpA1* transgenes. The protocol could be applied to adult flies with some modifications as ORNs can be activated by illuminating the antennae, except for the Or24a ORN, which is specific for larvae (Fishilevich et al., 2005; Kreher et al., 2005, 2008). Alternatively, red-shifted variants of ChR (Lin et al., 2013a,b; Inagaki et al., 2014) could be used to generate *Or-ChR*^*red*^ flies that can be activated by optogenetic tools. The fly stocks used in this protocol are made available from the Bloomington Stock Center.

## Author contributions

KF designed the project. TH and CL performed the experiments and optimized the protocol. KH constructed parts of the plasmid and examined the protocol. TH, KH, and KF wrote the manuscript. All authors reviewed the manuscript.

### Conflict of interest statement

The authors declare that the research was conducted in the absence of any commercial or financial relationships that could be construed as a potential conflict of interest.
