# Supplementary material for: Artificial Induction of Associative Olfactory Memory by Optogenetic and Thermogenetic Activation of Olfactory Sensory Neurons and Octopaminergic Neurons in Drosophila Larvae
Source: Front Behav Neurosci. 2016 Jun 28;10:137. doi: 10.3389/fnbeh.2016.00137 (PMC4923186; doi:10.3389/fnbeh.2016.00137)
Supplement: Supplementary file 1 [file SupplementalMaterials.PDF]

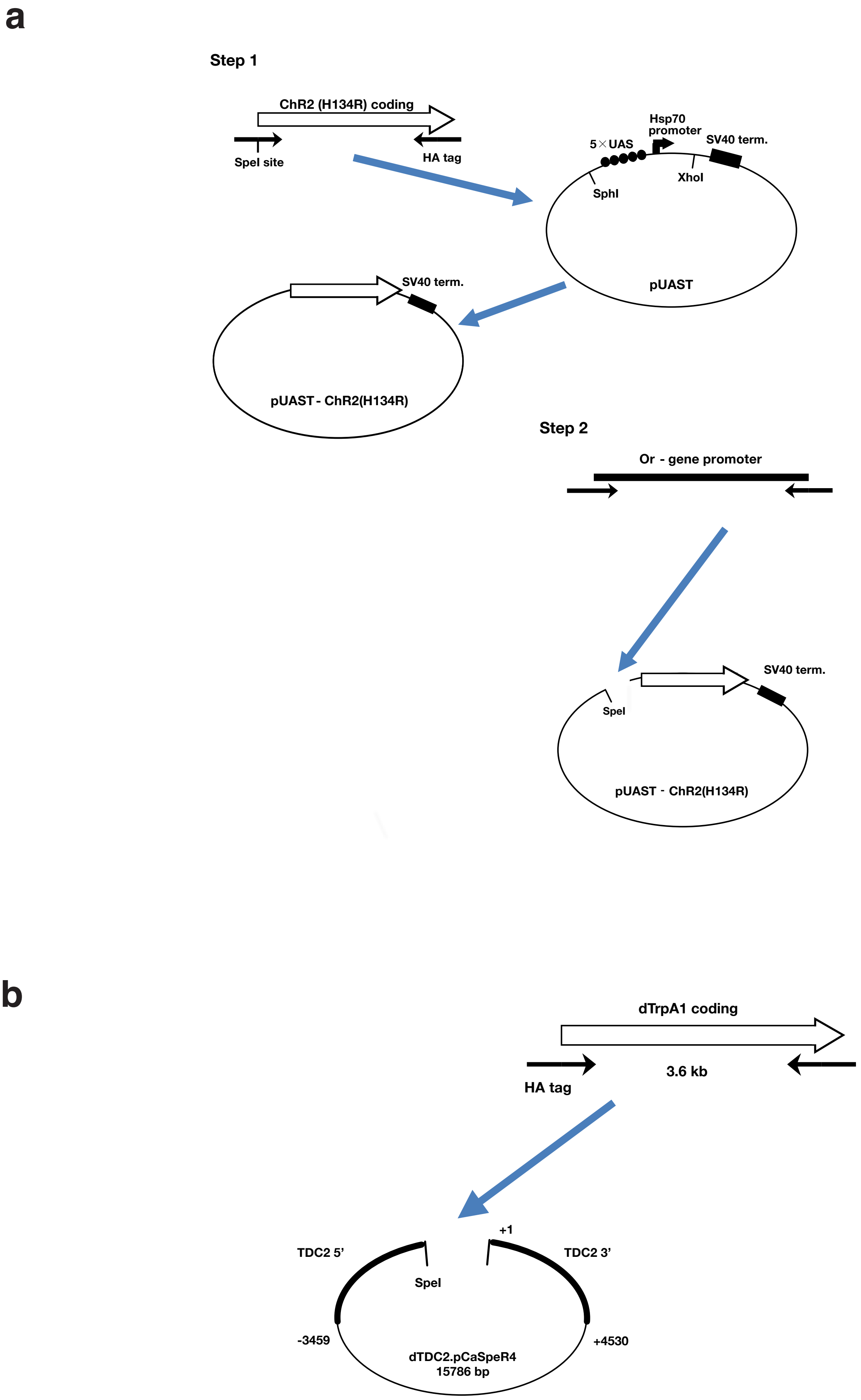

Supplemental Figure 1

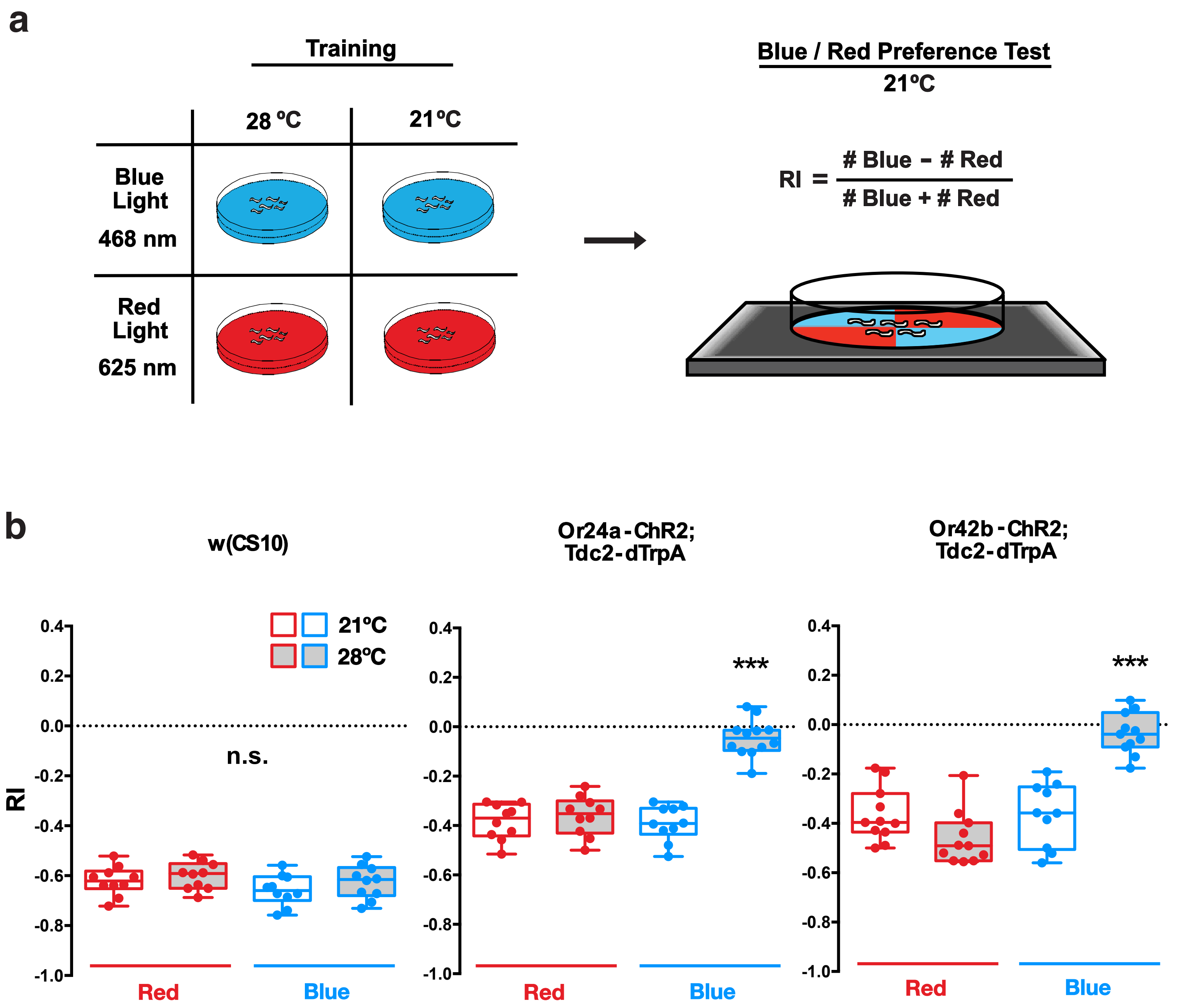

Supplemental Figure 2

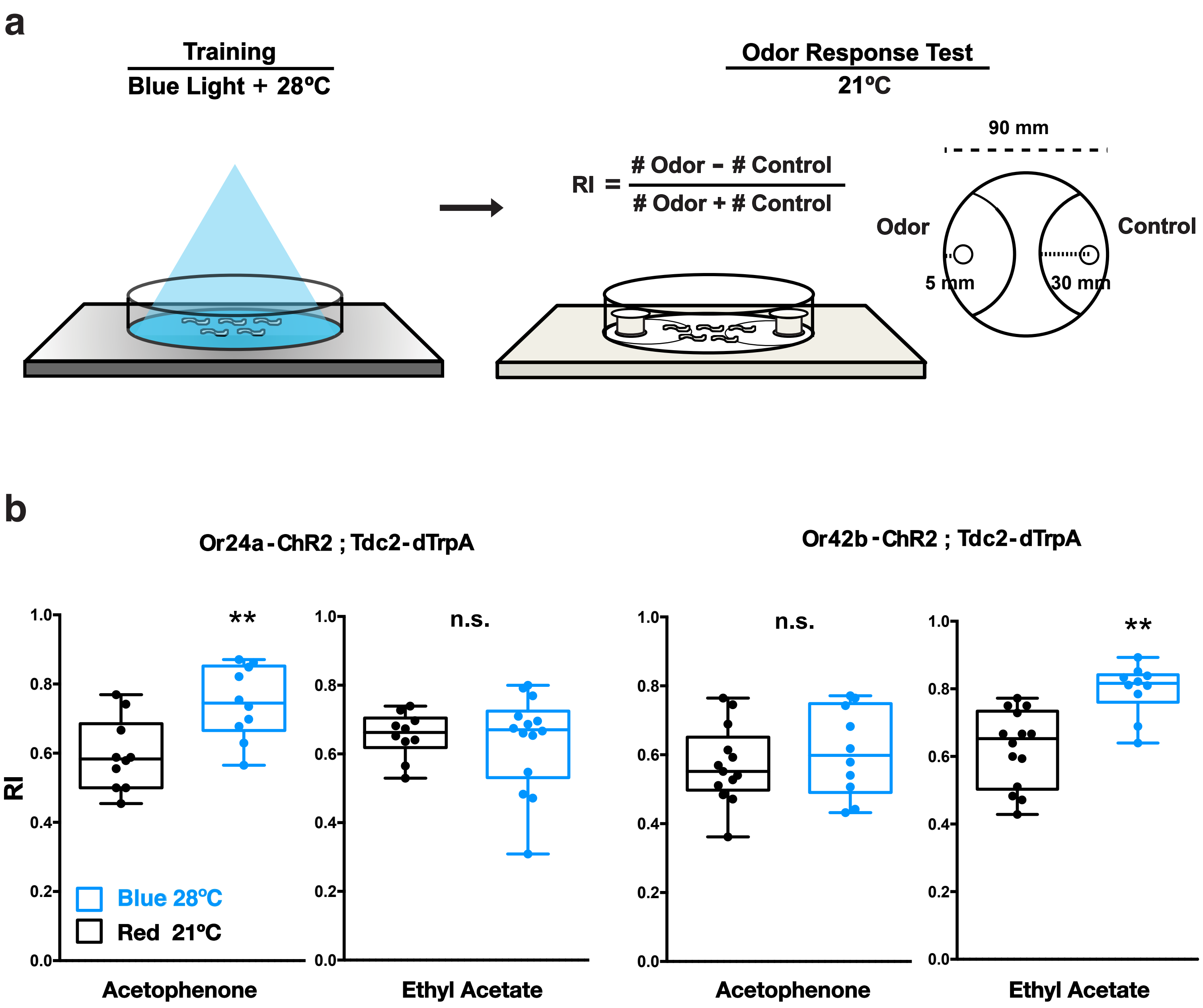

Supplemental Figure 3

Supplemental Figure 1

Plasmid construction

(a) Or-ChR2 constructs.

The Or promoter sequences were amplified based on the sequences used in the respective Or-GAL4 lines. The promoter sequence of Or83b (2642 base pairs) was amplified based on the sequence used in Or83b-GAL4 (Wang et al., 2003). The promoter sequences of Or42b (8039 base pairs), Or24a (8720 base pairs), and Or82a (1865 base pairs) were amplified based on the sequences used in the respective Or-GAL4 lines (Fishilevich et al., 2005)

We inserted the PCR-amplified ChR2 (H134R) coding fragment (open arrow) (a gift from Karl Deisseroth) tagged with HA into a P-element based pUAST vector by replacing the vector sequence (Sph I – Xho I) spanning 5x UAS and Hsp70 basic promoter with ChR2 coding fragment. The ChR2 fragment was amplified with In-Fusion oligonucleotides (solid arrows), which involve a Spe I site in the amino terminus and the HA sequence in the carboxyl terminus. The Or-promoter fragment was PCR-amplified with In-fusion oligonucleotides (solid arrows) and inserted at the Spe I site upstream of the ChR2 coding sequence on the derived plasmid.

(b) Tdc2-dTrpA1 construct.

Construction of Tdc2-dTrpA1 was done according to the scheme used to construct Tdc2-GAL4 (Cole et al., 2005). Genomic sequence containing a region from -3459 to 14530 of the dTdc2 locus was amplified through stepwise PCR and cloned into pCaSpeR4. A 3.6 kb DNA fragment containing the coding region of dTrpA1 (open arrow) (Hamada et al., 2008) (gift from Paul Garrity) was PCR-amplified with In-Fusion oligonucleotides with amino terminal HA tag (solid arrows), and then inserted upstream of the dTdc2 coding sequence (Cole et al., 2005).

We verified all the constructs by complete sequencing prior to germinal trans-formation, which was performed by Genetics Services, Inc. (Sudbury, MA).

Supplemental Figure 2

Light/heat controls : splitting conditioning as technical controls

(a) Larval conditionings and phototaxis test. Transgenic larvae carrying both *Or-ChR2* and *Tdc2-dTrpA1* were conditioned for 60 sec at 28 ºC or 21 ºC in combination with blue or red light. Larval phototactic behavior was tested at 21 ºC on the blue/red quadrants plate. Response index (RI) was calculated as indicated. (b) Phototactic responses of transgenic larvae differentially conditioned with blue light or heat. Only the combined stimulation with blue light and heat caused suppression of negative phototaxis toward blue light for the double transgenic larvae (*Or24a-ChR2; Tdc2-dTrpA1* and *Or42b-ChR2; Tdc2-dTrpA1*) while no difference was induced with red light at either temperature.

None of the conditionings altered the phototactic response of the *w (CS10)* control larvae. ***p < 0.001 Kruskal-Wallis test followed by Dunn's post-hoc test. Box plots represent the median as the middle line, 25th and 75th percentile as box boundaries, as well as the minimum and the maximum as whiskers, respectively. Data set was adapted from Honda et al., 2014.

Supplemental Figure 3

Olfactory responses test after artificial conditioning

(a) Olfactory response test. After the artificial conditioning with blue light and heat, larvae were transferred to an olfactory test plate at 21 ºC, on which the test odorant is spotted on one side and none on the other side. After 3 min, the number of animals moved in the indicated semicircular areas was counted. Response index (RI) was calculated as indicated. (b) Odor specificity of artificial memory. Black box/whisker plots: control (60 sec red light at 21 ºC). Blue box/whisker plots: associative conditioning (60 sec blue light at 28 ºC). Odorants used in the olfactory response test are indicated under the box. **p < 0.01 by Mann-Whitney *U*-test between 21 ºC and 28 ºC. n = 10-14 trials. Box plots represent the median as the middle line, 25th and 75th percentile as box boundaries, as well as the minimum and the maximum as whiskers, respectively. Data set was adapted from Honda et al., 2014.

References

Cole, S.H., Carney, G.E., Mcclung, C.A., Willard, S.S., Taylor, B.J., and Hirsh, J. (2005). Two functional but noncomplementing Drosophila tyrosine decarboxylase genes: distinct roles for neural tyramine and octopamine in female fertility. *The Journal of biological chemistry* 280**,** 14948-14955.

Fishilevich, E., Domingos, A.I., Asahina, K., Naef, F., Vosshall, L.B., and Louis, M. (2005). Chemotaxis behavior mediated by single larval olfactory neurons in Drosophila. *Current biology : CB* 15**,** 2086-2096.

Hamada, F.N., Rosenzweig, M., Kang, K., Pulver, S.R., Ghezzi, A., Jegla, T.J., and Garrity, P.A. (2008). An internal thermal sensor controlling temperature preference in Drosophila. *Nature* 454**,** 217-220.

Wang, J.W., Wong, A.M., Flores, J., Vosshall, L.B., and Axel, R. (2003). Two-photon calcium imaging reveals an odor-evoked map of activity in the fly brain. *Cell* 112**,** 271-282.
